# Back pain prevalence and associated factors in children and adolescents: an epidemiological population study

**DOI:** 10.1590/S1518-8787.2016050006175

**Published:** 2016-05-31

**Authors:** Matias Noll, Cláudia Tarragô Candotti, Bruna Nichele da Rosa, Jefferson Fagundes Loss

**Affiliations:** I Instituto Federal Goiano. Ceres, GO, Brasil; II Universidade Federal do Rio Grande do Sul. Porto Alegre, RS, Brasil

**Keywords:** Child, Adolescent, Back Pain, epidemiology, Risk Factors, Posture, Cross-Sectional Studies

## Abstract

**OBJECTIVE:**

To identify the prevalence of back pain among Brazilian school children and the factors associated with this pain.

**METHODS:**

All 1,720 schoolchildren from the fifth to the eight grade attending schools from the city of Teutonia, RS, Southern Brazil, were invited to participate in the study. From these, 1,597 children participated. We applied the Back Pain and Body Posture Evaluation Instrument. The dependent variable was back pain, while the independent one were demographic, socioeconomic, behavior and heredity data. The prevalence ratio was estimated by multivariate analysis using the Poisson regression model (α = 0.05).

**RESULTS:**

The prevalence of back pain in the last three months was 55.7% (n = 802). The multivariate analysis showed that back pain is associated with the variables: sex, parents with back pain, weekly frequency of physical activity, daily time spent watching television, studying in bed, sitting posture to write and use the computer, and way of carrying the backpack.

**CONCLUSIONS:**

The prevalence of back pain in schoolchildren is high and it is associated with demographic, behavior and heredity aspects.

## RESUMO

**OBJETIVO:**

Identificar a prevalência de dor nas costas de escolares brasileiros e os fatores associados a essa dor.

**MÉTODOS:**

Todas os 1.720 estudantes das escolas do quinto ao oitavo ano de todas as escolas de Teutônia, RS, foram convidados a participar do estudo. Desse total, 1.597 crianças participaram. Foi utilizado o questionário *Back Pain and Body Posture Evaluation Instrument*. A variável dependente foi a dor nas costas e as variáveis independentes foram os dados demográficos, socioeconômicos, comportamentais e hereditários. A razão de prevalência foi calculada pela análise multivariada feita pelo modelo de regressão de Poisson (a = 0,05).

**RESULTADOS:**

A prevalência de dor nas costas nos três últimos meses foi de 55,7% (n = 802). A análise multivariada demonstrou que a dor nas costas está associada com as variáveis de sexo, pais com dor nas costas, frequência semanal de exercício físico, tempo gasto por dia assistindo à televisão, estudar na cama, postura ao dormir, postura sentada para escrever e usar o computador, e o modo de carregar mochila.

**CONCLUSÕES:**

A prevalência de dor nas costas em escolares é alta e está associada com aspectos demográficos, comportamentais e hereditários.

## INTRODUCTION

In global terms, back pain is a common complaint in industrialized societies, with chronic back pain reportedly affecting between 54.0% and 90.0% of the adult population^7^. It is considered a public health problem and is the most common complaint among workers in all fields. Back pain has been shown to have a negative impact on overall health, and it results in personal and social disruption. The National Health Survey (2013), conducted by the Brazilian Ministry of Health together with the Brazilian Institute of Geography and Statistics (IBGE)^a^, showed that 27 million adults in the country are affected by chronic disease in spine, which corresponds to 18.5% of the adult population in Brazil^6^. In addition to being widespread among adults^13^, back pain is also reported in childhood and adolescence, frequently presenting in two or more anatomic areas of the spine in young schoolchildren^18^.

Back pain in schoolchildren has multiple causes, including physical, behavioral, genetic, and psychosocial factors^8^. Hence, sex, age, physical exercise, length and quality of regular sleep, depression and anxiety, family history of back pain, educational level of parents and time spent watching television, using the computer, playing videogames, and seated have been identified as risk factors for developing back pain in schoolchildren^1^ ^,^ ^2^ ^,^ ^6^ ^,^ ^9^ ^-^ ^11^ ^,^ ^24^.

Empirically, health professionals also consider the body postures adopted during daily activities a risk factor for the occurrence of back pain, although few studies report this in literature^2^. Two recent studies (Meziat Filho et al.^12^ and Pereira et al.^19^) that attempted to assess the association between postural habits (watching television and using the computer) and back pain confirmed that back pain is highly prevalent, has a substantial impact on late adolescence, and is associated with inappropriate home postural habits. However, we notice methodological concerns in the development of the questionnaires used, as well as doubts regarding their reproducibility and validation procedures: (1) unclear description of the instrument construction, that is, whether it was analyzed by experts or if it was developed based only on the experience of the outside researchers involved; (2) no information on the sample in the test and re-test procedures; (3) poor quality illustrations; and (4) only moderate reliability^12^ or no information provided regarding the reliability^19^ of the questionnaire.

Nevertheless, there remains a lack of research evaluating the relation between back pain and other postures such as sleeping posture, sitting posture to write, posture when sitting on a bench, posture adopted to lift objects from the floor, and means used to transport school material and mode of transporting one’s school bag^15^ ^,^ ^16^.

The recurring and increasing incidence of back pain generates significant costs for governments, making the implementation of reforms and preventative care programs necessary^7^. From this perspective, it is important to be aware of the factors that contribute to back pain in order to develop effective preventative strategies. In spite of being widespread in the literature, the risk factors remain controversial, mainly related to body postures adopted during daily activities^2^ ^,^ ^8^ ^,^ ^15^. Still, it is known that the collected data cannot be extrapolated into different contexts, since sociocultural, environmental, and genetic influences that are unique to each locality contribute to the condition. Therefore, the aims of this study were to (1) identify the prevalence of back pain in basic schoolchildren in an epidemiological population study and (2) identify which factors are associated with back pain.

## METHODS

We conducted an epidemiological population cross-sectional study. All 1,720 basic schoolchildren from the fifth to eighth grades (from 11 to 16 years of age) in all 11 schools of Teutonia, RS, Southern Brazil, were invited to participate in the study. From this total, 1,597 schoolchildren participated; 7.2% (n = 123) refused to participate or missed school on the day of the evaluations. The frequency and percentage of schoolchildren stratified by sex and age are presented in Table 1.

**Table 1:** Frequency and percentage of schoolchildren evaluated stratified by sex and age.

Age (years)	Male		Female		Total	
		
	n	%	n	%	n	%
11	99	11.6	107	14.4	206	12.9
12	192	22.4	181	24.4	373	23.4
13	197	23.0	179	24.2	376	23.5
14	203	23.7	172	23.2	375	23.5
15	120	14.0	83	11.2	203	12.7
16	45	5.3	19	2.6	64	4.0
Total	856	100	741	100	1,597	100

To verify the prevalence of back pain and behavioral and postural habits, we used a self-administered questionnaire, the Back Pain and Body Posture Evaluation Instrument (BackPEI). It is a valid and reproducible questionnaire, consisting of 21 closed questions and a different version for each sex^16^. The BackPEI addressed the following issues: (1) back pain in the last three months (occurrence and frequency); (2) demographics (age and sex); (3) socioeconomics (parental education and type of schooling); (4) behavioral (physical activity, read or study in bed, time per day spent watching television and using computer, time per day sleeping); (5) postural factors (sitting posture to write, to use computer and to talk, way of carrying school supplies, and sleeping posture); and (6) heredity (occurrence of back pain in parents).

For the question about the occurrence of back pain, “Have you felt (or have been feeling) back pain in the last three months?”, those who responded “I don’t know”, which is an important option to avoid bias from the obligation to choose “yes” or “no”, were excluded from all subsequent analyses. Moreover, only those schoolchildren who self-reported back pain in the last three months answered the question about frequency of back pain. The same was true for the questions about physical exercise, since only those who reported practicing physical exercise answered the questions about frequency and competitive physical exercise. Furthermore, only those who reported using a backpack answered the question about the way of carrying school material.

The questions related to sitting posture when writing, using a computer, and talking, as well as the postures adopted when lifting an object from the floor and when carrying school materials, were composed of figures showing subjects performing the activities. Each question had five or six alternatives, including the “Another way/I don’t know” alternative. Only one alternative was considered the correct way to perform each activity; the remaining alternatives were grouped as inadequate in the statistical analysis^16^.

The reproducibility of the questions, which were analyzed using the kappa (*k*) coefficient, was classified as ‘very good’ (*k* > 0.8) or ‘good’ (0.6 < *k* < 0.8). Besides the risk factors commonly evaluated in prevalence studies, the BackPEI also includes a photograph-based evaluation of the key postural habits of school-age children and has two versions, one for each sex, thereby facilitating the identification of school-age children with the content of each question, their interpretation of the question and, consequently, a more representative response^16^.

All schools were invited to participate in this study by a meeting with the City Department of Education (CDE/Teutonia), in which the research aims and collection procedure were explained. After obtaining agreement from the CDE/Teutonia, a meeting was scheduled with the principal of each school to present the research project. When agreement was obtained from all principals, we scheduled the evaluations for each school. The researcher responsible for administering the questionnaire handed a BackPEI copy to each student in the classroom. The researcher explained to all students how the questionnaire should be answered.

The Statistical Package for the Social Sciences version 20.0 was used for the statistical analysis. The age variable was grouped by two-year intervals (11 and 12 years, 13 and 14 years, and 15 and 16 years). Data were analyzed using descriptive statistics and the Chi-square test of association (bivariate analysis) for the dependent variable back pain, with demographics, socioeconomics, behavioral, postural, and heredity variables as independent variables. The independent variables with a significance level of p < 0.20 in the bivariate analysis were included in the Poisson regression model with robust variance. The measure of effect used was the prevalence ratio with their respective 95% confidence intervals (95%CI) (α = 0.05).

The present study was conducted in accordance with the Helsinki Declaration and was approved by the Ethics Research Committee from Universidade Federal do Rio Grande do Sul (Process 19832).

## RESULTS

The prevalence of back pain in the last three months was 55.7% (n = 802). Among all schoolchildren (n = 1,597), 158 chose the alternative “I don’t know” and were excluded from this analysis. The results also showed different data for male and female participants, with a higher percentage of girls reporting back pain. The figure presents back pain prevalence in the last three months stratified by sex and age.

**Figure:**
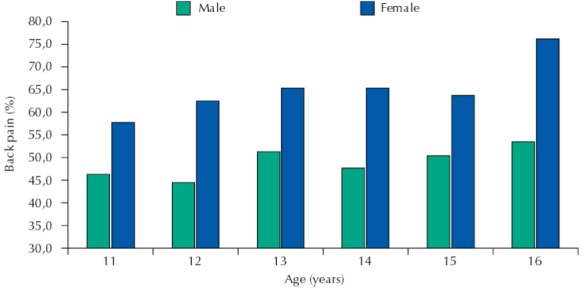
Results of back pain prevalence in the last three months among schoolchildren stratified by sex and age.

Regarding pain frequency, most schoolchildren reported feeling back pain only once in the last three months (29.8%) or once a month (40.4%). Also, 15.5% of the students reported that back pain prevented them from performing daily activities such as playing, studying, and participating in sports. Table 2 presents the descriptive data by sex, both for back pain frequency and for hindrance of daily activities.

**Table 2:** Back pain frequency in the last three months and impediment of performing daily activities.

Variable	Male		Female		Total	
		
	n	%	n	%	n	%
Frequency						
Only once	126	34.1	112	26.0	238	29.8
Once a month	135	36.6	188	43.6	323	40.4
Once a week	32	8.7	47	10.9	79	9.9
Two to three times per week	25	6.8	35	8.1	60	7.5
Four times or more per week	18	4.9	17	3.9	35	4.4
No answer	33	8.9	32	7.5	65	8.0
Impediment of performing activities of daily living						
Yes	40	10.8	84	19.5	124	15.5
No	311	84.1	323	74.9	634	79.1
No answer	19	5.1	24	5.6	43	5.4

The results related to demographics, socioeconomics, and hereditary factors are presented in Table 3. Those related to behavioral factors are presented in Table 4. Bivariate analysis showed that back pain is associated with sex and parents with back pain (Table 3) as well as weekly frequency of physical exercise, time spent per day watching television, reading or studying in bed, sleeping posture, sitting posture to write, sitting posture to use computer, and the way of carrying one’s backpack (Table 4). Multivariable analysis confirms the bivariate results.

**Table 4:** Association (χ2) and prevalence ratio between dependent variable back pain and independent variables (behavioral).

Variable	n	%	Back pain (n)	Back pain (%)	χ^2a^	Prevalence ratio	95%CI
Behavioral							
Physical exercise (n = 1,437)							
Yes	1,287	89.6	727	56.5	0.103	1	
No	150	10.4	74	49.3		0.95	0.9–1.01
Physical exercise weekly frequency (n = 1,295)^d^							
Zero day per week	150	11.6	74	49.3	0.003^b^	1	
One to two days per week	627	48.4	382	60.9		1.07	1.01–1.14
Three; four days per week	357	27.6	181	50.7		1.01	0.94–1.07
Five or more days per week	161	12.4	85	52.8		1.02	0.95–1.11
Practice competitive exercise (n = 1,273)^c^							
Yes	560	44.0	302	53.9	0.128	1	
No	713	56.0	415	58.2		1.02	0.99–1.06
Time spent watching television per day (n = 1,229)							
Zero; three hours per day	698	56.8	359	51.4	0.001^b^	1	
Four; seven hours per day	421	34.2	252	59.9		1.05	1.01–1.09
Eight or more hours per day	110	9.0	77	70.0		1.12	1.06–1.18
Time spent using computer per day (n = 1,102)							
Zero; three hours per day	735	66.7	407	55.4	0.536	1	
Four; five hours per day	231	21.0	132	57.1		1.01	0.96–1.06
Six or more hours per day	136	12.3	82	60.3		1.03	0.97–1.09
Time sleeping per night (n = 1,225)							
Zero; seven hours per day	409	33.4	239	58.4	0.449	1	
Eight; nine hours per day	624	50.9	341	54.6		0.97	0.93–1.01
Ten or more hours per day	192	15.7	105	54.7		0.97	0.92–1.03
Read or study in bed (n = 733)							
No	285	38.9	144	50.5	0.021^b^	1	
Yes	448	61.1	266	59.4		1.05	1.01–1.11
Postural							
Sleeping posture (n = 1,308)							
Supine	103	7.9	44	42.7	0.005^b^	1	
Lateral decubitus	838	64.1	458	54.7		1.08	1.01–1.16
Prone	367	28.0	222	60.5		1.12	1.04–1.21
Sitting posture; write (n = 1,401)							
Adequate	213	15.2	100	46.9	0.008^b^	1	
Inadequate	1,188	84.8	678	57.1		1.06	1.01–1.12
Sitting posture on a bench (n = 1,394)							
Adequate	183	13.1	93	50.8	0.126	1	
Inadequate	1,211	86.9	690	57.0		1.04	0.98–1.09
Sitting posture; use computer (n = 1,394)							
Adequate	301	21.6	150	49.8	0.018^b^	1	
Inadequate	1,093	78.4	630	57.6		1.05	1.01–1.09
Posture; lift object from the floor (n = 1,339)							
Adequate	105	7.8	67	63.8	0.069	1	
Inadequate	1,234	92.2	680	55.1		0.94	0.89–1.04
Carrying school supplies (n = 1,439)							
Backpack	1,332	92.6	749	56.2	0.190	1	
Another (briefcase, purse, and others)	107	7.4	53	49.5		0.95	0.89–1.02
Way; carry backpack (n = 1,320)^c^							
Adequate (symmetrical handles on both shoulders)	1,166	88.3	639	54.8	0.019^b^	1	
Inadequate (asymmetric)	154	11.7	99	64.3		1.06	1.01–1.11

^a^ Bivariate analysis. Wald Chi-square test.

^b^ Significant association (p < 0.05).

^c^ Related only to those schoolchildren to which the variable applies.

^d^ Those containing the response “I don’t know, it depends on the week” were excluded.

**Table 3:** Association (χ2) and prevalence ratio between dependent variable back pain and independent variables (demographics, socioeconomics, and hereditary factors).

Variable	n	%	Back pain (n)	Back pain (%)	χ^2a^	Prevalence ratio	95%CI
Demographics							
Sex (n = 1,439)							
Male	765	53.2	371	48.5	0.001^b^	1	
Female	674	46.8	431	63.9		1.11	1.06–1.14
Age (n = 1,439)							
11 and 12 years	513	35.6	273	53.2	0.363	1	
13 and 14 years	682	47.4	390	57.2		1.02	0.98–1.06
15 and 16 years	244	17.0	139	57.0		1.02	0.97–1.07
Socioeconomics							
Education network (n = 1,439)							
@ State	569	39.5	327	57.5	0.43	1	
Town	745	51.8	403	54.1		0.97	0.94–1.01
Private	125	8.7	72	57.6		1	0.94–1.06
Mother education (n = 1,151)							
Did not attend school	8	0.7	3	37.5	0.357	1	
Elementary school	699	60.7	398	56.9		1.14	0.89–1.45
High school	336	29.2	194	57.7		1.14	0.89–1.46
College degree	108	9.4	69	63.9		1.19	0.92–1.53
Father education (n = 1,097)							
Did not attend school	13	1.2	8	61.5	0.974	1	
Elementary school	677	61.7	391	57.8		0.97	0.82–1.15
High school	306	27.9	173	56.5		0.96	0.82–1.14
College degree	101	9.2	58	57.4		0.97	0.81–1.16
Hereditary							
Parents with back pain (n = 1,174)							
No	447	38.1	170	38.0	0.001^b^	1	
Yes	727	61.9	491	67.5		1.21	1.16–1.26

^a^ Bivariate analysis. Wald Chi-square test.

^b^ Significant association (p < 0.05).

## DISCUSSION

According to this transversal epidemiological population study, we conclude that elementary schoolchildren present a high incidence of back pain. From the multivariable analysis, back pain is more prevalent in females, those with a low weekly frequency (one to two days a week) of physical exercise, children whose parents also present with back pain, and those who spend long periods (more than eight hours per day) watching television, reading or studying in bed, and adopting an improper posture when performing daily activities, including sleeping, sitting to write or use the computer, and carrying school materials.

The results showed a high prevalence of back pain among this population. Indeed, the prevalence found here is between back pain rates described in the literature, which vary from 20.0% to 70.0%^22^ ^-^ ^24^. In a study by Skoffer^22^ investigating the frequency of low back pain in 546 schoolchildren aged between 14 and 17 years from a city in Denmark, 51.3% indicated feeling low back pain in the three months previous to the research. Of these, 24.2% reported that the pain resulted in sleep disorders and required specialized medical care. Similar results were found in the present study, in which 15.5% of schoolchildren reported that back pain prevented them from performing daily activities, affecting more girls (19.5%) than boys (10.8%). Further, approximately 40.0% reported back pain once a month (Table 2). Although the occurrence of pain was high, the frequency of pain was low, perhaps because the subjects were children and adolescents. However, early interventions are needed (i.e., in the school environment), in such a way that in the long term a change might be brought about the current situation, where 18.5% of Brazilian adults are affected by chronic disease in the spine.

Kovacs et al.^8^ assessed the prevalence of back pain in 7,048 teenagers from Mallorca, Spain, and found a high prevalence, which was higher for females (69.3%) than males (50.9%). This pain also restricted the activities of a higher proportion of girls (30.7%) than boys (21.0%)^8^. The figure shows that females reported a higher incidence of feeling back pain than males, corroborating many studies^17^ ^,^ ^20^ ^,^ ^21^. A possible explanation for these results may be the earlier maturity of females and their anatomical and functional characteristics (shorter, less muscle, and bone density) in relation to males. Moreover, it has been reported that it is more socially acceptable for women to show their symptoms and feelings because of both societal and educational factors^21^ ^,^ ^24^.

Although the present study did not find a significant association between back pain and age, the literature has shown an increase in both back pain prevalence and back pain incidence relative to age^24^. A review by Balagué et al.^2^ provides evidence of a substantial increase in the back pain odds ratio with age, increasing from 2.79 in the 10-12-year age group to 16.5 in the 16-20-year age group. In the present study, we found an apparent trend towards an increased prevalence of back pain with increased age, but it was not statistically significant.

Regarding physical exercise, no significant association was found between physical activity and back pain (Table 4). This result was unexpected, as the beneficial effects of physical activity on pain are well-documented^23^ ^,^ ^25^. However, regarding the frequency of physical exercise, the multivariable analysis showed an apparently contradictory result. On one hand, those who exercise one to two days a week are more likely to experience pain than those who exercise more than three days a week, which could indicate a decrease in pain with an increase in the weekly frequency of exercise. On the other hand, those who do not exercise are also less likely to experience pain than those who exercise from one to two days a week, which could indicate that not exercising is better than doing exercise at a low frequency.

These results do not allow us to endorse the findings in the literature suggesting that practicing exercise is a protective factor against back pain. This was the case in Skoffer and Foldspang’s^23^ study; although they could not indicate which physical activities were effective in preventing back pain, they verified that back pain in teenagers was associated with physical inactivity. In a study conducted by Wedderkopp et al.^25^, children who performed high-intensity physical exercise had a lower prevalence of back pain after three years than did those who did not exercise at the same intensity. Our study did not investigate the types of physical activity. As improper practice of an activity can cause or aggravate pain^23^, we understand that this discussion requires further investigation.

Another relevant finding, although little investigated thus far, is the association between back pain in parents or the responsible guardian and back pain in schoolchildren. Table 3 indicates that schoolchildren whose parents or responsible guardian presented with back pain had a higher likelihood of also presenting with back pain. It is speculated that this association is due to not only genetic factors, but also behavioral and psychosocial factors^2^. From this perspective, assuming that a consequence of chronic back pain in family life is interference with a child’s pain etiology, it is believed that schoolchildren whose parents present back pain are accustomed to hearing complaints from parents, and become more inclined to report back pain themselves^2^ ^,^ ^6^.

This study also showed a relation between back pain and the time spent watching television. Table 4 shows that watching television for more than eight hours per day is a risk factor for back pain. Similarly, Vitta et al.^5^ showed that 71.1% of the students who watched television more than two hours per day had twice the probability of having back pain. It is also believed that these facts can explain the significant association between presenting back pain and improperly sitting while writing and using the computer found in this study. Students who remain seated for long periods throughout the day, much of the time in an inappropriate posture (forward trunk flexion, lack of lumbar support, and lack of forearm support), are predisposed to higher levels of general discomfort, such as pain, fatigue, tingling in different parts of the body, and especially degenerative processes such as disc herniation. Act of sitting can increases overhead compression on intervertebral discs, and extended periods in the sitting position can (1) lead to disc malnutrition^18^ contributing to the development of general discomfort; and (2) initiate mechanisms that can endanger the integrity of the musculoskeletal system^11^ ^,^ ^12^ ^,^ ^18^.

A significant relationship was found between the occurrence of back pain and inadequate sitting posture when writing and when using a computer. Also related to postural habits, Table 4 shows the significant association between back pain and reading or studying in bed and sleeping in an inadequate position. In another words, schoolchildren who engage in these positions have a higher chance of developing back pain. To accomplish the task of reading or studying in bed, as well as inadequate sitting posture when writing and when using a computer, schoolchildren can support an awkward posture (lordosed or kyphosed, overly arched or slouched), resulting in increased intradiscal pressure, which may harm the spine. Moreover, supporting these postures has been described as a risk factor for developing back pain^11^. Regarding sleeping posture, the recommended sleep positions are supine and lateral decubitus, and any other positions can result in imbalanced loads on the intervertebral discs and the facet joints. Sleeping in a position other than those recommended can compromise the disc hydration that occurs during sleep, as this hydration directly depends on the amount of pressure and on the way in which this is applied above the intervertebral discs.

A non-neutral lying posture can lead to lateral bending of the spine and unbalanced loading on intervertebral discs and facet joints, which may produce injuries in these structures^10^. In addition, postural changes affect the relative orientation between adjacent vertebrae and alter the load distribution between the apophyseal joints and intervertebral discs. Therefore, the manner in which a person sleeps, sits, or moves can affect the pain perception from innervated tissues, although that load may be insufficient to produce an injury. The pain mechanism can thus be referred as a functional pathology. Thus, to some extent, these factors may explain the significant association between back pain and inadequate sleeping posture.

Table 4 shows a significant association between the presence of back pain and the way school supplies are carried. In addition to considering the weight of the backpack, which should ideally not exceed 10.0% of the child’s body weight, we must also pay attention to the way school supplies are carried, as carrying backpacks in an asymmetric way is associated with back pain, as already described in the literature^22^. This result can be associated with the torque-side slope generated on the spine when the backpack is carried above only one shoulder, and is significantly reduced when the backpack is carried above both shoulders^24^. Despite having found a significant association with the improper carrying of school materials, some limitations in the present study related to this subject should be noted such as not weighing the school materials and not verifying the time spent carrying school materials. The risk factors for musculoskeletal discomfort related to carrying school materials are known to include the combined effects of heavy loads, backpack shape and size, and time spent carrying school supplies^2^ ^,^ ^3^.

Moreover, given that the assessment of postural habits depends on the interpretation of the photograph by the evaluated respondent, this instrument outstands from others because it was developed with separate versions for boys and girls, which facilitates the access to its content by other researchers. Considering that the questionnaire format is widely used in descriptive and epidemiological studies, the BackPEI may be applied in studies designed to evaluate back pain and its associated factors, particularly bad posture in daily activities in school-age children^16^. However, even though the BackPEI^16^ contains the “I don’t know” alternative in all questions, in order to avoid any bias arising from the obligation to choose a specific alternative, it still has the limitation of being a self-administered and self-referred questionnaire, which makes it an interpretation-dependent instrument^15^.

According to the array of evidence presented, the importance of a “Back School” program in the school context is needed, once the numerous surrounding risk factors in developing back pain are understood^3^ ^,^ ^14^. Considering that the literature already shows the positive effects of Back School postural habits when performing daily activities^4^, such initiatives get to the root of the problem and directly intervene on several risk factors^9^.

## Footnotes

a Instituto Brasileiro de Geografia e Estatística. National survey of health 2013: perceived health status, lifestyles and chronic diseases Brazil, major regions and federation units. Rio de Janeiro (RJ): Instituto Brasileiro de Geografia e Estatística; 2014.
